# A single session of exercise increases connectivity in sensorimotor-related brain networks: a resting-state fMRI study in young healthy adults

**DOI:** 10.3389/fnhum.2014.00625

**Published:** 2014-08-14

**Authors:** Ahmad S. Rajab, David E. Crane, Laura E. Middleton, Andrew D. Robertson, Michelle Hampson, Bradley J. MacIntosh

**Affiliations:** ^1^Department of Medical Biophysics, University of TorontoToronto ON, Canada; ^2^Heart and Stroke Foundation Canadian Partnership for Stroke Recovery, Sunnybrook Research InstituteToronto, ON, Canada; ^3^Department of Kinesiology, University of WaterlooWaterloo, ON, Canada; ^4^Department of Diagnostic Radiology, Yale University School of MedicineNew Haven, CT, USA

**Keywords:** BOLD fMRI, aerobic exercise, single session effect, resting-state networks, independent component analysis (ICA), sensorimotor, denoising, functional connectivity

## Abstract

Habitual long term physical activity is known to have beneficial cognitive, structural, and neuro-protective brain effects, but to date there is limited knowledge on whether a single session of exercise can alter the brain’s functional connectivity, as assessed by resting-state functional magnetic resonance imaging (rs-fMRI). The primary objective of this study was to characterize potential session effects in resting-state networks (RSNs). We examined the acute effects of exercise on the functional connectivity of young healthy adults (*N* = 15) by collecting rs-fMRI before and after 20 min of moderate intensity aerobic exercise and compared this with a no-exercise control group (*N* = 15). Data were analyzed using independent component analysis, denoising and dual regression procedures. Regions of interest-based group session effect statistics were calculated in RSNs of interest using voxel-wise permutation testing and Cohen’s D effect size. Group analysis in the exercising group data set revealed a session effect in sub-regions of three sensorimotor related areas: the pre and/or postcentral gyri, secondary somatosensory area and thalamus, characterized by increased co-activation after exercise (corrected *p* < 0.05). Cohen’s D analysis also showed a significant effect of session in these three RSNs (*p*< 0.05), corroborating the voxel-wise findings. Analyses of the no-exercise dataset produced no significant results, thereby providing support for the exercise findings and establishing the inherent test–retest reliability of the analysis pipeline on the RSNs of interest. This study establishes the feasibility of rs-fMRI to localize brain regions that are associated with acute exercise, as well as an analysis consideration to improve sensitivity to a session effect.

## INTRODUCTION

Exercise research has traditionally focused on the effects of physical activity on the cardiovascular and musculoskeletal system. Over the last decade, however, there has been a burgeoning body of literature on the positive effects of exercise on the brain [see Thomas et al. (2012) for a review]. While animal literature provides the framework to study the neurobiology of exercise (Black et al., 1990; van Praag et al., 1999; Markham and Greenough, 2004; Pereira et al., 2007), there are still many fundamental questions about exercise in humans. Human studies show that regular exercise can produce structural brain changes (Colcombe et al., 2006), may reverse age-related decline (Erickson et al., 2011) and can contribute to positive cognitive changes (Kramer et al., 1999; Hillman et al., 2008).

The bulk of this exercise neuroimaging literature involves long term exercise; however, less is known about the single session effects that accumulate to produce long term benefits. Electroencephalographic studies have shown that a single session of maximal intensity aerobic exercise produces changes in the brain’s alpha and beta band activity (Moraes et al., 2007; Schneider et al., 2009). Furthermore, a meta-analysis reported that cognitive performance is positively impacted by acute exercise (Chang et al., 2012). In the current study, we attempt to establish resting-state functional magnetic resonance imaging (fMRI) as a neuroimaging marker that can be used to study how exercise impacts the brain.

As demonstrated by Gusnard et al. (2001), the brain “at rest” is far from resting. While resting-state functional neuroimaging was first studied using positron emission tomography (Raichle et al., 2001), blood oxygenation level-dependent (BOLD) contrast imaging has proven itself to be a powerful and popular technique for studying the brain’s resting activity. Rs-fMRI is appealing because of high temporal and spatial resolution, ease of use, non-invasive assessment, and its potential role as a biomarker (Filippini et al., 2009). Additionally, the existence of on-going massive data-sharing initiatives (Biswal et al., 2010) provides further proof of the potential and popularity of fMRI for mapping brain activity. First demonstrated by Biswal et al. (1995), the BOLD signal exhibits spontaneous, coherent and correlated temporal oscillations (0.01-0.1 Hz) across different brain regions even in the absence of external stimuli or a goal-directed task. The term “resting-state networks” (RSNs) thus refers to spatially distinct, functionally relevant networks that each possess different temporal characteristics. RSNs have since been shown to exist across species (Vincent et al., 2007) and populations (Damoiseaux et al., 2006), and their functional connectivity can be modulated by disease (Fox and Greicius, 2010), states of consciousness like sleep (Fukunaga et al., 2006) and interventions like pharmacological studies (Khalili-Mahani et al., 2012).

Interventions designed to impact brain health, such as an exercise program, have recently started to consider rs-fMRI as a tool to understanding the related brain changes (Voss et al., 2010). While it is yet to be proven, establishing that it is feasible to study exercise session effects using rs-fMRI will open up a new angle from which to study the brain-exercise relationship. The non-invasive and task-independent natures of rs-fMRI make it suitable for most clinical populations, and its good spatial resolution and amenability to different analysis techniques make it a good approach for studying session effects.

Thus, the primary objective of this study was to determine whether a single bout of aerobic exercise produces a measurable change in functional connectivity in a cohort of healthy, young adults. We used independent component analysis (ICA) because it is a robust and reliable technique (Zuo et al., 2010) used to parcellate rs-fMRI data into neuro-anatomically relevant RSNs, is conducive to an exploratory approach and also because it enables the separation of data into components of interest versus established artefactual components. We hypothesized that exercise-sensitive RSNs would show a change in their activity after exercise. Our secondary objective is to validate our findings by evaluating the test–retest reliability of our methods, using a no-exercise rs-fMRI dataset. Advancing our understanding of the immediate effects of exercise by developing novel neuroimaging analytical methods may help to explain how short term effects contribute to long term brain changes.

## MATERIALS AND METHODS

### PARTICIPANTS AND STUDY DESIGN

#### Exercise dataset

Sixteen young healthy adults (10 women) between the ages of 20 and 35 years were recruited for this study. One MRI dataset was corrupted and consequently this participant was excluded from analysis (see **Table 1**). Exclusion criteria included contraindications to MRI or inability to complete the exercise session. The Sunnybrook Health Sciences Centre Research Ethics Board approved this study and all participants provided signed informed consent. Detailed below, the study protocol entailed: a baseline pre-exercise MRI scan, a 25-min exercise session, a 10-min no-exercise cool down period and a post-exercise MRI scan.

**Table 1:** Participant demographics.

Characteristics		Exercise		No-exercise	
N		15		15
Ages (years)		26.1 ± 4.3		27.0 ± 6.5
Sex (M/F)		6/9		8/7
Handedness (L/R)		3/12		0/15

#### No-exercise dataset

A second dataset was included in this study to act as an exercise null scenario, and used to help validate exercise findings (see **Table 1**). MRI data were accessed from the Neuroimaging Informatics Tools and Resources Clearinghouse (NITRC) database (http://www.nitrc.org/frs/?group_id=296; dataset *NewHaven_b*). This dataset included multi-session rs-fMRI in a cohort of 16 healthy (7 women) adults between the ages of 18 and 42, with comparable MRI scan parameters to the exercise dataset. One dataset was randomly removed so that this group matched the exercise group. Although four rs-fMRI sessions were available for each subject, we restricted our analysis to sessions 1 and 2, as to more closely match the exercise data since they preceded an actual task in the scanner.

### SINGLE EXERCISE SESSION

Exercise was performed on a semi-recumbent cycle ergometer. Participants spent 2 min cycling to warm up, 20 min exercising at 70% (moderate intensity) of their age-predicted maximum heart rate (HR), and finally 3 min cooling down at a self-determined pace. We used the age-predicted maximum HR, defined as 220 bpm minus age in years (Londeree and Moeschberger, 1984), to determine the exercise target HR. HR was monitored in real-time using a Polar heart rate monitor and participants were given verbal feedback if they deviated from their target HR. During exercise, power output (watts) and pedaling frequency (rpm) were recorded every minute, and ratings of perceived exertion (RPE; self-reported using a 10-point Borg scale) were recorded every 10 min. Systolic and diastolic blood pressure (mmHg) were recorded before and 10 min following exercise.

### MAGNETIC RESONANCE IMAGING

#### Exercise dataset

Magnetic resonance imaging data were acquired on a Philips 3T Achieva MRI scanner equipped with an 8-channel head coil. Rs-fMRI BOLD data were acquired pre- and post-exercise. A T1 anatomical image was also acquired post-exercise for registration purposes. The imaging parameters were: T_2_∗-weighted echo planar imaging (TR/TE = 1500/30 ms, FA = 70^∘^, 80 × 80 × 28 matrix, voxel = 3.0 mm × 3.0 mm × 5.0 mm, 28 slices, 230 volumes, acquisition time = 6:00 min:s), T1-weighted structural (TR/TE/TI = 9.5/2.3/1400 ms, FA = 8, 256 × 164 × 140 matrix, voxel = 1.0 mm × 1.2 mm × 1.2 mm, acquisition time = 8:38 min:s). Participants were instructed to refrain from drinking caffeine an hour before scanning and to keep their eyes open during the resting-state scans.

#### No-exercise dataset

Magnetic resonance imaging data were acquired on a Siemens 3T scanner within a single session and without an exercise intervention. Scan parameters were: EPI BOLD (TR/TE = 1500/25 ms, FA = 80, 64 × 64 × 22 matrix, voxel = 3.4 mm × 3.4 mm × 5.5 mm, 22 slices, 181 volumes, acquisition time = 4:31 min:s), T1-weighted structural (TR/TE/TI = 2000/3.67/1100 ms, FA = 7^∘^, 256 × 256 × 160 matrix, voxel = 1.0 mm × 1.0 mm × 1.0 mm, acquisition time = 6:24 min:s). Participants were instructed to keep their eyes open during the rs-fMRI scans.

### MRI ANALYSIS

Analysis was conducted using FMRIB Software Library (FSL) tools (Jenkinson et al., 2012) and in-house scripts. *Structural images* were processed using FSL’s brain extraction tool (BET; Smith, 2002), and visually inspected for optimal extraction. *Functional images* were analyzed using FSL’s Multivariate Exploratory Linear Optimized Decomposition into Independent Components (MELODIC) tool (Beckmann et al., 2005). Automatic dimensionality estimation (Laplace) was used for all MELODIC analyses. Data were analyzed using two pipelines: (1) an unsupervised analysis approach (Raw), and (2) a visually inspected structured artifact removal (VISTAR) noise suppression approach.

#### Raw pipeline

Data were pre-processed: motion corrected, high pass filtered, slice time corrected, spatially smoothed (5 mm FWHM using a Gaussian kernel), linearly registered to each individual’s structural image and then a 3 mm MNI template. Group-level MELODIC analysis identified several RSNs. Given the paucity of rs-fMRI exercise literature, we focused on well established RSNs including those potentially related to motor activity. Additionally, two other RSNs were selected, the default mode network (DMN) RSN, for its perceived clinical utility (Schwindt et al., 2013), and the medial visual RSN, as a control. A dual regression technique (Filippini et al., 2009) was used to produce participant/session-specific RSNs. RSN time courses were variance normalized as part of the dual regression procedure. The value at each voxel in these participant/session-specific RSN images represented regression coefficients. To investigate resting-state co-activation differences in the RSNs between sessions, these resulting subject-specific regression coefficient images were compared with a paired *t*-test across the group in a voxel-wise analysis and corrected for multiple comparisons using FSL’s randomize (5000 permutations, threshold-free cluster enhancement; Nichols and Holmes, 2002). Voxel-wise analyses were restricted to supra-threshold group RSN z-statistics maps as a means to restrict the session effect analysis to within-network voxels. This procedure was done for each of the RSNs under investigation. A *post hoc* test was conducted for RSNs that showed a significant session, whereby the mean heart rate during the rs-fMRI scan was included as a covariate of non-interest. To characterize each RSN as a whole and test for a session effect, a second method was used based on the subject-specific regression coefficient images (i.e., stage two dual regression outputs). A Cohen’s D effect size was calculated to quantify session differences based on a significant non-zero effect across the group. Analysis was again limited to voxels circumscribed by the group RSN volume.

#### VISTAR pipeline

Pre-processing steps were carried out in the same manner as in the Raw pipeline, but with the addition of a subject-level MELODIC. These subject-specific MELODIC outputs were inspected manually and ICs of non-interest (IC_noise_). IC_noise_ were removed by spatial regression using a tool in FSL (i.e., fsl_regfilt). These components of non-interest consisted of head motion, eye motion, CSF signal, high frequency signals and other spurious signals as described in a previous MELODIC study (Kelly et al., 2010). A single author (ASR), trained to identify spurious signal in rs-fMRI, performed the manual inspection. The VISTAR datasets were then used in a group-level MELODIC, and RSNs of interest were again identified. Dual regression and permutation testing, as well as Cohen’s D analysis were repeated. This analysis was also limited to group level RSN masks. A spatial correlation coefficient was used to identify comparable RSNs between Raw and VISTAR pipelines.

## RESULTS

### EXERCISE DATASET

#### Single session exercise

The average age and body mass index of the 15 exercising participants (9 women) were 26.1 ± 4.1 years and 22.5 ± 2.1 kg/m^2^_,_ respectively. HR during the moderate intensity exercise session was 70 ± 3.5 % of age-predicted maximum. The average RPE was 3.9 ± 1.4, after 10 min of exercise and 4.5 ± 1.8 after 20 min; both of which fall in the descriptive range of “somewhat strong” to “strong.” Systolic blood pressure remained significantly higher than baseline at 10 min post-exercise (baseline: 112 ± 12 mmHg, post: 118 ± 13 mmHg, *p* = 0.007). Diastolic blood pressure, however, was not significantly different (baseline: 74 ± 9 mmHg, post: 73 ± 8 mmHg, *p* = 0.544). The mean time between the end of exercise and the second rs-fMRI scan was 16:51 ± 3:33 min:s. Finally, HR was significantly higher at the start of the post-exercise rs-fMRI scan (baseline: 70 ± 12 bpm, post: 78 ± 9 bpm, *p* < 0.005).

#### Raw pipeline

**FIGURE 1:**
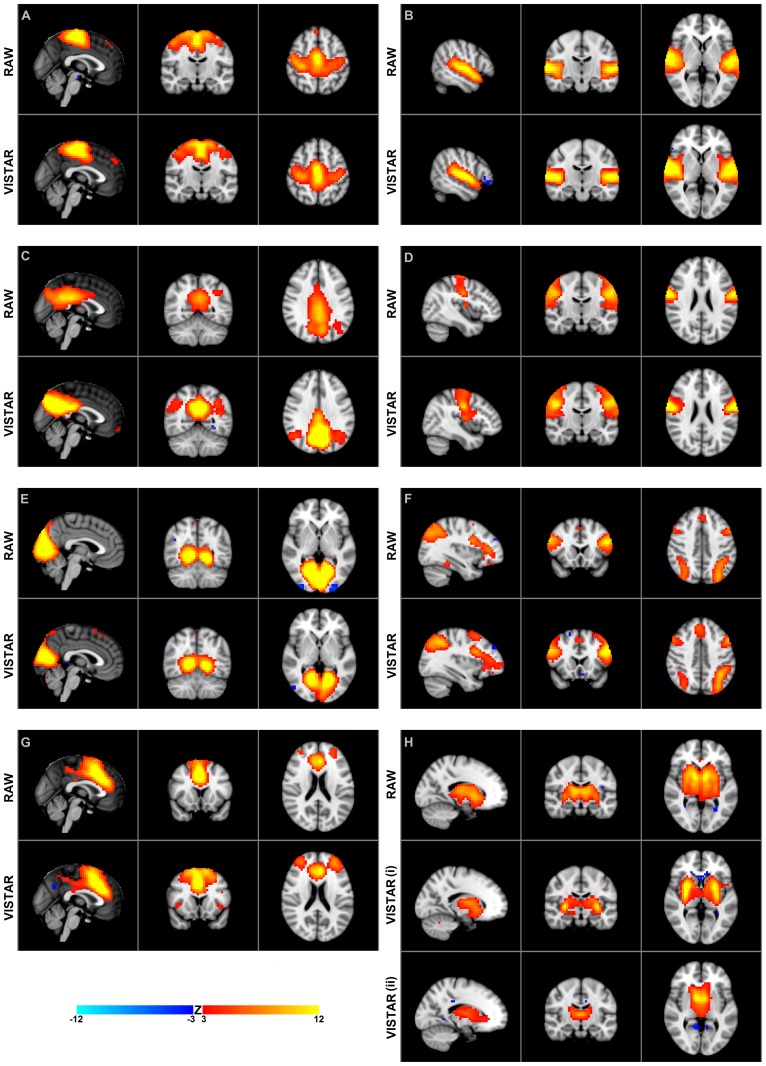
**Group independent component analysis (ICA) maps using temporal concatenation.** The Raw pipeline produced eight spatial maps and the visually inspected structured artifact removal (VISTAR) pipeline produced nine, with the basal ganglia being split into two components. Both pipelines produce spatially similar maps scc, spatial cross correlation. **(A)** sensori motor resting-state networks (RSN): scc = 0.88, MNI (0,-14,52); **(B)** auditory RSN: scc = 0.88, MNI (54,-16,2); **(C)** default mode network RSN: scc = 0.79, MNI (0,-56,32); **(D)** pre/post central gyri RSN: scc = 0.85, MNI (42,-14,24); **(E)** medial visual RSN: scc = 0.88, MNI (2,-58,0); **(F)** attention RSN: scc = 0.80, MNI (-34,18,42); **(G)** executive RSN: scc = 0.89, MNI (0,14,20); **(H)** basal ganglia RSN: scc = 0.77 (i) 0.67 (ii) MNI (-16,-14,2).

The total number of group MELODIC components for the exercise dataset was 46 and 43, Raw and VISTAR, respectively. Using group level ICA, we identified the following eight RSNs: (a) sensorimotor, (b) auditory, (c) default mode (DMN), (d) pre/post central gyri, (e) medial visual, (f) attention, (g) executive, and (h) basal ganglia (see **Figure 1**). Voxel-wise analysis showed no effect of session after cluster-enhancement and correction for multiple comparisons. Cohen’s D analysis obtained borderline insignificance in the auditory, pre/post central gyri and basal ganglia RSNs (*p* = 0.075, *p*= 0.081, *p*= 0.117, respectively), while the other five RSNs showed no significant effect size (*p* > 0.264).

#### VISTAR pipeline

**FIGURE 2:**
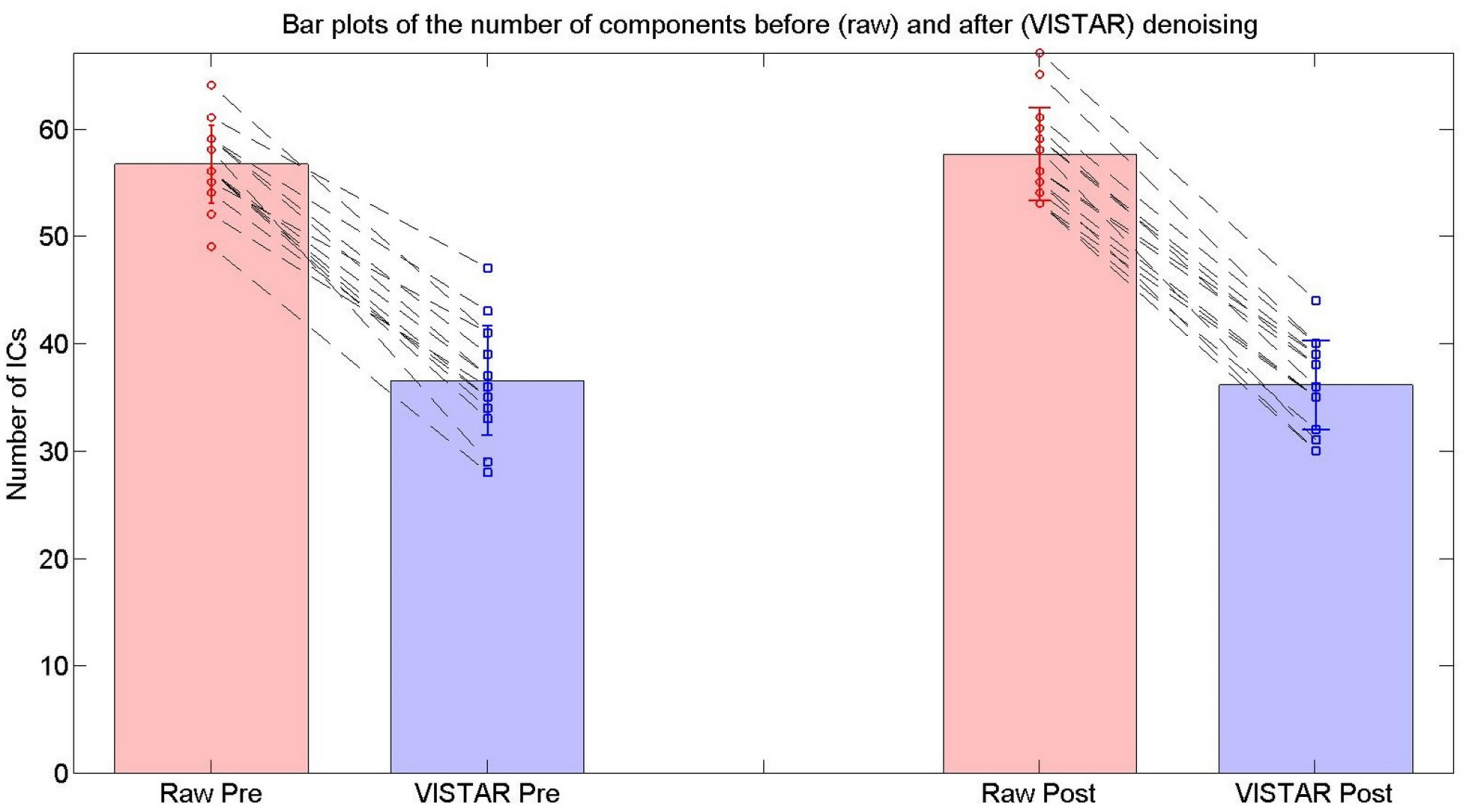
**The number of ICs used for the RSN group analyses in the exercise dataset from the Raw and VISTAR pipelines.** No significant differences were noted in the number of ICs pre- vs. post- Raw (*p*= 0.274), or pre- vs. post- VISTAR (*p*= 0.747).

**FIGURE 3:**
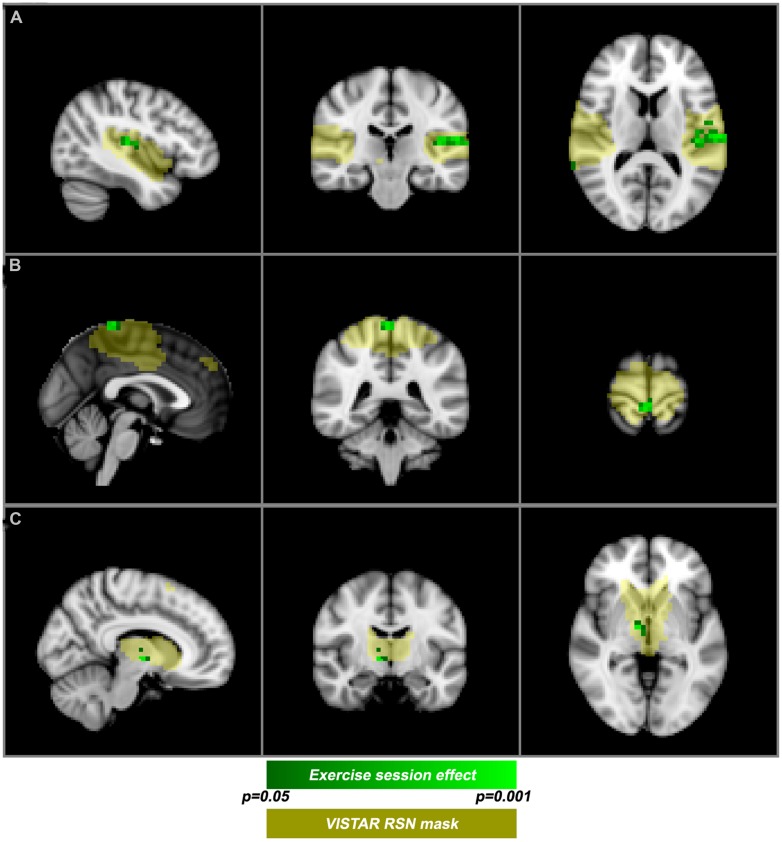
**Post-exercise changes in resting-state functional magnetic resonance imaging functional magnetic resonance imaging (rs-fMRI).** VISTAR RSNs (yellow) overlaid with cluster enhanced-regions of increased co-activation (green): **(A)** auditory RSN [MNI(-42,-24,12)], **(B)** sensorimotor RSN [MNI (0,-40,72)], **(C)** Thalamic-Caudate RSN [MNI (10,-10,-2)].

Visually inspected structured artifact removal removed 36% of a total 1714 ICs (see **Table 2**). There was no significant difference in the number of ICs produced using single-session ICA pre- vs. post-exercise (*p* = 0.274), or the number removed pre- vs. post-exercise (*p* = 0.223) by session (see **Figure 2**). Group level ICA produced nine RSNs – the same previous eight identified by the Raw pipeline, with the exception of the basal ganglia, which was split in two components: putamen and thalamic/caudate RSNs (see **Figure 1**). There was good spatial overlap between Raw and VISTAR RSNs (spatial overlap: 0.82 ± 0.07; see **Figure 1**). Three RSNs were identified as showing voxel-wise session differences (*p*_corrected_ < 0.05), with clusters in the following regions (see **Figure 3**): (1) the auditory RSN showed increased co-activation in the central and parietal operculum cortices, (2) the sensorimotor RSN showed increased co-activation in the pre and/or postcentral gyri, and (3) the thalamic-caudate RSN shows increased activity in the right thalamus. The results of Cohen’s D RSN-based regions of interest (ROI) analysis revealed that the auditory, sensorimotor and thalamic-caudate networks exhibited a significant session effect (*p* = 0.014, *p*= 0.049, *p*= 0.023, respectively; see **Table 3**). The pre/post central gyri RSN showed a trend (*p* = 0.059), while all other RSNs were not significant (*p* > 0.428). Including a heart rate covariate in the paired design did not influence the session findings for the auditory RSN (*p*_corrected_ < 0.05), but did impact the thalamic-caudate and pre-post central gyri RSN (*p*_corrected_ > 0.18).

**Table 2:** Details of VISTAR IC removal.

Characteristics		Exercise		No-exercise
Total number of ICs		1714		2032
Total number of artifact ICs removed		624		625
Mean number of ICs per participant per session (SD)		57 ± 4		68 ± 3
Mean number of artifact ICs per participant per session (SD)		21 ± 4		21 ± 5
Min/Max % of removed artifact ICs per session		23/50		18/48

**Table 3:** Cohen’s D effect size comparing pre- vs. post- (exercise) and session 1 vs. 2 (no-exercise) using the VISTAR Pipeline.

	Exercise dataset		No-exercise dataset	
RSN	Mean ± SD	*p*-value	Mean ± SD	*p*-value
Sensorimotor	0.18 ± 0.33	0.049*	0.04 ± 0.27	0.591
Auditory	0.18 ± 0.25	0.014*	0.07 ± 0.41	0.545
DMN	0.04 ± 0.19	0.480	0.02 ± 0.22	0.756
Pre/post central gyri	0.13 ± 0.25	0.059	0.02 ± 0.55	0.875
Medial visual	0.05 ± 0.26	0.428	0.05 ± 0.38	0.651
Attention	0.00 ± 0.21	0.957	-0.03 ± 0.32	0.765
Executive	0.02 ± 0.27	0.832	-0.08 ± 0.27	0.244
Putamen	0.04 ± 0.36	0.661	0.03 ± 0.40	0.796
Caudate/thalamus	0.22 ± 0.34	0.023*	0.11 ± 0.39	0.308

^*^p < 0.05.

### NO-EXERCISE DATASET

The average age of the 15 participants (7 women) was 27.0 ± 6.5 years. There was no significant difference in ages between the exercise and no-exercise subjects (*p*= 0.791).

#### Raw pipeline

The total number of group MELODIC components for the no-exercise dataset was 59 and 50, for the Raw and VISTAR, respectively. Group level ICA produced the same nine RSNs as the exercise dataset when analyzed with the VISTAR pipeline. Voxel-wise analysis showed no effect of session after cluster-enhancement and correction for multiple comparisons, while Cohen’s D analysis showed a session-effect in the executive RSN (*p* = 0.009).

#### VISTAR pipeline

**FIGURE 4:**
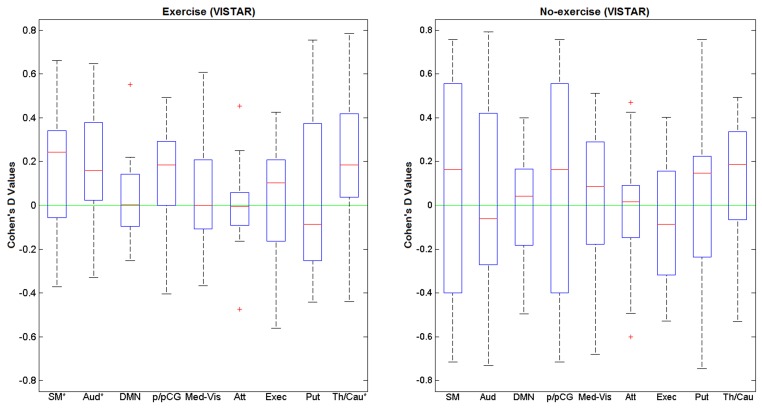
**Cohen’s D values of for the nine RSNs of interest using exercise (*N* = 15) and no-exercise (*N* = 15) datasets (VISTAR).** SM, sensorimotor; Aud, auditory; DMN, default mode network; p/pCG, pre and post central gyri; Med-Vis, medial visual; Atten, attention; exec, executive; Put, putamen; Th/Cau, thalamus and caudate.

Visually inspected structured artefact removal removed 31% of a total number of 2032 ICs (see **Table 2**). There was no significant difference in the number of ICs produced using single-session ICA session 1 vs. 2 (*p*_corrected_ = 0.595), or the number removed from session 1 vs. 2 (*p*= 0.529; see **Figure 2**). Group level ICA produced the same nine RSNs as in the Raw pipeline. There was good spatial overlap between Raw and VISTAR for eight RSNs (spatial overlap: 0.80 ± 0.14). No significant session differences were identified (*p* > 0.329). Cohen’s D analysis found no significant session effect size (*p*> 0.244; see **Figure 4** and **Table 3** for a comparison between these results and the exercise results). The spatial correlation coefficient between the respective RSN patterns for exercise and no-exercise datasets were 0.61 ± 0.17 for the Raw pipeline and 0.62 ± 0.14 for the VISTAR pipeline (note: the standard deviation values were derived from the variation between RSNs).

## DISCUSSION

This study demonstrates the feasibility of using rs-fMRI to detect and localize acute effects of exercise on brain connectivity. We observed a change in the resting-state BOLD functional connectivity of young healthy adults in three RSNs, predominantly localized to cortical areas involved in sensorimotor activity. The exercise effect was sensitive to voxel-wise and Cohen’s D analyses, but only after the data were processed with a denoising procedure. Our methods also demonstrated good test–retest reliability using the no-exercise control dataset.

The central and parietal operculum cortices, commonly referred to as the secondary somatosensory region (S2), showed increased co-activation after exercise. The S2 region has been shown to modulate tactile attention (Burton et al., 1999). Increased co-activation observed in the current study was primarily lateralized to the left S2. In addition, a cortical area responsible for processing motor function and tactile sensation from the lower limbs also exhibited an increase in co-activation. This area forms part of the motor and sensory homunculi, and the sensorimotor RSN generated by ICA. A previous PET study (Christensen et al., 2000) demonstrated that an analogous region of the motor homunculus exhibited increased activity during active cycling. Finally, we observed an exercise-related effect in a basal ganglia RSN, specifically in the thalamus. This RSN is of interest given the role that the basal ganglia play in motor learning and reward (Alexander et al., 1990), but to date has not been reported in structural MRI exercise-brain studies (Colcombe et al., 2006; Erickson et al., 2011). Previous studies report that aerobic exercise has an effect on cognition (Yanagisawa et al., 2010) and attention (Kamijo et al., 2004) brain networks. We observed that the auditory RSN had increased co-activation after acute exercise, on the basis of the Cohen’s D effect size analysis. It is conceivable that other regions besides the parietal and central operculum may have contributed toward this session difference. In this context, we note that Patriat et al. (2013) have reported that the auditory RSN can be influenced by subtle differences in the resting-state instructions (e.g., eyes open vs. eyes closed).

To our knowledge, this is the first study that examined changes in rs-BOLD functional connectivity after a single session of exercise and helps to establish the feasibility of probing acute brain effects of exercise. A novel aspect of this study was the use of the Cohen’s D to assess the session differences within an entire RSN. This procedure detected an effect of session in the same three aforementioned RSNs. While Cohen’s D does not localize sub-regions within an RSN, it is much less computationally intensive than permutation testing and proved to be sensitive to a session effect. This approach may be conducive to compare longitudinal changes in RSNs of interest, like the DMN (Greicius et al., 2004; Tanabe et al., 2011; Schwindt et al., 2013).

Another important contribution of this study is the added value of “denoising” rs-fMRI data as a means to increase the sensitivity of our analysis to detect exercise effects. Artifact ICs (i.e., ICs of non-interest) are well-established; however, few tools exist for automatically detecting ICs of non-interest [see Murphy et al. (2013) for a review of denoising techniques and Salimi-Khorshidi et al. (2014) for a recent semi-automated denoising tool]. For this study, each dataset was decomposed using MELODIC into ICs, and each IC’s spatial and temporal signatures were manually inspected. The manual inspection approach is time-consuming (i.e., approximately 10–30 min to evaluate ICs from one dataset); however, visual inspection is still the gold standard for artifact detection when using ICA.

This study is not without its limitations. First at the time of the repeat rs-fMRI scan, the heart rate decreased after exercise but was nonetheless still significantly greater than the pre-exercise heart rate. Using heart rate as a covariate in the paired design group analysis did influence the session-related findings for two of the three significant RSNs. Blood pressure was not measured continuously so we cannot rule out the possibility that it too was elevated at the time of the repeat rs-fMRI scan. Second, the no-exercise dataset was not an identical match to the exercise protocol but was selected because it was closely matched the age and scan parameters. Performing the comparable analyses on both datasets helped contextualize our findings. Third, recent work (Power et al., 2012) demonstrates that head motion can influence the resting-state connectivity inference, even after standard motion correction. To address this possibility, *post hoc*, we reviewed the head motion summary metrics derived from the motion coregistration procedure. We found no significant session differences when considering relative or absolute head motion data (*p* > 0.33). As to be expected, however, a *post hoc* test revealed that there was a significant correlation between the number of IC_noise_ attributed to head motion and the head motion summary metrics (*p* < 0.005).

Systolic blood pressure was elevated post-exercise, while breathing rate and arterial blood O_2_ and CO_2_ levels were not monitored. Although we cannot rule out the possibility that differences in global vascular regulation during the post-exercise scans contributed to the altered functional connectivity patterns, our VISTAR approach was designed to remove physiological noise and thus minimize these potential mitigating physiological effects. However, the specificity of our finding to motor areas of the brain aligns with our previous work on cerebral blood flow changes (MacIntosh et al., 2014), in support of the notion that exercise can lead to a neural carryover effect.

Future work is needed to investigate the effect that exercise parameters, like type, duration and intensity, as well as baseline fitness levels (i.e., via cardiopulmonary exercise test), have on acute exercise responses. Additionally, although outside the scope of the current study due to sample size considerations, age, sex, and genetics have been shown to affect baseline functional connectivity (Biswal et al., 2010; Glahn et al., 2010), and investigating their influence on exercise effects will further our understanding of the scope of the exercise–brain relationship. Further work is also required to establish the optimal time post-exercise to best observe functional connectivity changes. Lastly, while this study established within-RSN session differences additional work is required to develop methods sensitive to inter-RSN session changes. In this study we restricted our analysis to within-network session differences, as opposed to between-network effects, because of the following considerations: (1) the need to be parsimonious in our analysis, given the relatively small sample size, (2) the more stringent correction for multiple comparisons when more voxels are considered (i.e., between-network situation). Future studies within a large sample may be more amenable to probe between-network effects that pertain to aerobic exercise. Future studies with a larger sample may be more amenable to probe between-network effects that pertain to aerobic exercise. In conclusion, these results suggest that rs-fMRI can be used to assess changes in functional connectivity that may relate to an acute exercise session.

## Conflict of Interest Statement

The authors declare that the research was conducted in the absence of any commercial or financial relationships that could be construed as a potential conflict of interest.
